# Prognostic relevance of prospero homeobox 1 and metastasis tumor antigen 1 in patients with malignant salivary gland tumors: a clinicopathological study

**DOI:** 10.1186/s40001-025-02863-2

**Published:** 2025-07-14

**Authors:** Samar Soliman, Doaa Abdallah Farag, Heba Ahmed Elhendawy

**Affiliations:** Oral Pathology, Faculty of Dentistry, Mansoura University, Mansoura, Egypt

**Keywords:** Salivary gland carcinomas, PROX1, MTA1, Immunohistochemistry, Distant metastasis, DFS, OS

## Abstract

**Objective:**

The current study aimed to investigate the prognostic relevance of PROX1, and MTA1 in salivary gland carcinomas.

**Methods:**

In a retrospective study on 45 cases diagnosed with salivary gland carcinoma, PROX1 and MTA1 immunoexpressions were assessed concerning the different clinicopathologic parameters, disease-free (DFS), and overall survivals (OS). Pearson’s Chi-square test, One-way ANOVA, and Post Hoc tests were used to estimate the difference between groups. The Kaplan–Meier method was used to record DFS and OS, and the log-rank test was used to analyze the data. With Cox regression, univariate and multivariate survival analyses were run. A *P*-value of 0.05 or less was regarded as statistically significant.

**Results:**

Positive PROX1 and high MTA1 expressions were significantly associated with large tumor sizes (T3 & T4), presence of nodal and distant metastasis, advanced TNM clinical stage (III + IV), and presence of tumor recurrence (*P* values were ≤ 0.05). Moreover, positive PROX1, and high MTA1 expressions were significantly associated with poor DFS and OS in the univariate models. Additionally, DFS and OS were significantly reduced in relation to large sized tumors (T3 + T4), positive nodal involvement, positive distant metastasis, advanced TNM clinical stage (III + IV), high-grade carcinomas, presence of LV invasion, and old ages (*P* values were ≤ 0.05). The multivariate analysis with Cox regression found that distant metastasis was the independent predictor for DFS, but not for OS in SGC patients.

**Conclusions:**

PROX1 and MTA1 immunoexpression could be used as predictors of progression and recurrence in SGC patients. PROX1 and MTA1 are potentially prognostic, predictive biomarkers, and promising molecular therapeutic targets in SGC patients.

*Trial registration* Retrospectively registered.

## Introduction

Salivary gland carcinomas (SGCs) is heterogeneous group, exhibiting varying morphological patterns and clinical behaviors for example mucoepidermoid carcinoma, adenoid cystic carcinoma, carcinoma ex pleomorphic adenoma, salivary duct carcinoma etc. [1]. Mucoepidermoid carcinoma (MEC) and adenoid cystic carcinoma (AdCC) are considered the most common ones [2]. Gland carcinoma (SGC) is a relatively rare disease of the head and neck that frequently presents distant metastases (DM) [3]. DM is considered one of the causes of treatment failure and death in SGCs. High-grade tumors have a greater chance for DM and poor prognosis, which range from 20 to 40% [4, 5]. As malignant salivary gland tumors are histologically heterogeneous groups with variable biological behavior, establishing a standard treatment for DM is a therapeutic challenge [6].Therefore, it is crucial to look for novel, reliable, and specific biomarkers to screen targeted therapies and predict prognosis precisely.

Prospero-related homeobox 1 (PROX1) gene is a transcription factor that is involved in the creation of lymphatic capillaries during embryonic development [7]. Also in the central nervous system, heart, lens, retina, liver, and pancreas [8].Various investigations have found that PROX1 not only serves as a tumor suppressor but also has oncogenic activity depending on the kind of cancer [7]. Furthermore, numerous investigations have found unusually high PROX1 expression in a variety of systemic malignant tumors, including neuroblastoma, colon cancer, kidney cancer, gastric cancer, esophageal squamous cell carcinoma, and hepatocellular carcinoma [7, 9]. Elevated PROX1 expression in individuals with neurocytoma was associated with a greater ratio of undifferentiated cells, an increased likelihood of lymphatic metastasis, and a poor prognosis [9].

Tumor-induced lymphangiogenesis and angiogenesis are very crucial for the development of cancer metastases [8, 10]. Many studies have reported that PROX1 is involved in lymphangiogenesis and angiogenesis in some types of tumors, such as gastric cancer [11], oral squamous cell carcinoma [12] and hepatocellular carcinoma [13].

Metastasis-associated genes (MTAs) are a gene family with three subtypes: MTA1, MTA2, and MTA3 [14]. MTA1 is the most basic member of the MTA family, and it was discovered as an upregulated gene using differential screening of a cDNA library from rat metastatic breast tumors [15]. It regulates the epithelial-mesenchymal transition (EMT), which is a key element in invasion and metastasis [16, 17]. MTA1 overexpression has been linked to a poor prognosis in malignant tumors, as well as increased invasion and metastasis [17, 18].

The role of prognostic indicators in SGCs is still debated in the English literature. As a result, the current work was designed to analyze the expression of PROX1 and MTA1 in SGCs and correlate their expression with several clinicopathological features. Furthermore, the incidence of recurrence and death was recorded, and patients'DFS and OS were calculated and analyzed in relation to the different levels of PROX1 and MTA1 expression to see if PROX1 and MTA1 immunoexpression could be used as predictors of progression and prognosis in SGC patients.

## Material and methods

### Patients’ selection and data retrieval

The current study was approved by the faculty of dentistry, Delta university ethical committee and it was conducted on 45 cases of salivary gland cancer; 13 tissue blocks of high-grade mucoepidermoid carcinoma (MEC), 12 blocks of adenoid cystic carcinoma (AdCC), 8 blocks of carcinoma ex pleomorphic adenoma (CXPA) representing high-grade SGC, and 7 blocks of low-grade MEC and 5 blocks of acinic cystic carcinoma (ACC) representing low-grade SGC according to the WHO classification of head and neck tumors criteria (2017) [19] collected from general pathology department, Oncology Center, Faculty of Medicine, Mansoura University and oral pathology department, Faculty of Dentistry, Mansoura University. Control group, five blocks of normal salivary gland tissue present in mucocele cases obtained from the archive of 2022 of oral pathology department, Faculty of Dentistry, Mansoura University. All the available clinical data for the studied cases were collected from patients registered medical documents from 2018 to 2019 in oral pathology department and the oncology center regarding the patient age, sex, site of the tumor, tumor size, presence or absence of lymph node metastasis, distant metastasis, and follow-up records for patients (Table 1). The cases included were primary tumors that were surgically treated and had complete follow-up records (at least for 3 years) (inclusion criteria). Cases with missing follow-up information and small-sized tissue samples were excluded (exclusion criteria). Following the end of the treatment, the patients’ follow-up began. The disease-free survival (DFS) and overall survival (OS) of the patients were gathered from their medical records.

**Table 1:** Clinicopathological characteristics of 45 cases diagnosed with salivary gland carcinoma

Clinicopathologic variables	No. (%)
Patients gender	
Male	18 (40%)
Female	27 (60%)
Age group	
< 65 years	19 (42.2%)
≥ 65 years	26 (57.8%)
Tumor site	
Parotid salivary gland	23 (51.1%)
Submandibular salivary gland	12 (26.7%)
Soft + hard palate minor salivary glands	5 (11.1%)
Sublingual major salivary gland	4 (8.8%)
Other sites	1 (2.2%)
Tumor size groups	
T1 and T2	19 (42.2%)
T3 and T4	26 (57.8%)
Nodal involvement	
Positive	26 (57.8%)
Negative	19 (42.2%)
Incidence of metastasis	
Present	17 (37.7%)
Absent	28 (62.3%)
TNM clinical stage	
Stage I and II	13 (28.8%)
Stage III and IV	32 (71.2%)
Tumor type	
Low grade MEC	7 (15.5%)
Acinic cell carcinoma	5 (11.1%)
High grade MEC	13 (28.8%)
Adenoid cystic carcinoma	12 (26.7%)
Carcinoma ex pleomorphic adenoma	8 (17.8%)
Histologic grade	
Low grade carcinomas	12 (26.7%)
High grade carcinomas	33 (73.3%)
Lymph vascular invasion	
Present	25 (55.6%)
Absent	20 (44.4%)
Incidence of recurrence	
Present	17 (37.8%)
Absent	28 (62.2%)
Incidence of death	
Died	5 (11.1%)
Alive	40 (88.9%)

Frequency table

### Immunohistochemistry

**Fig. 1:**
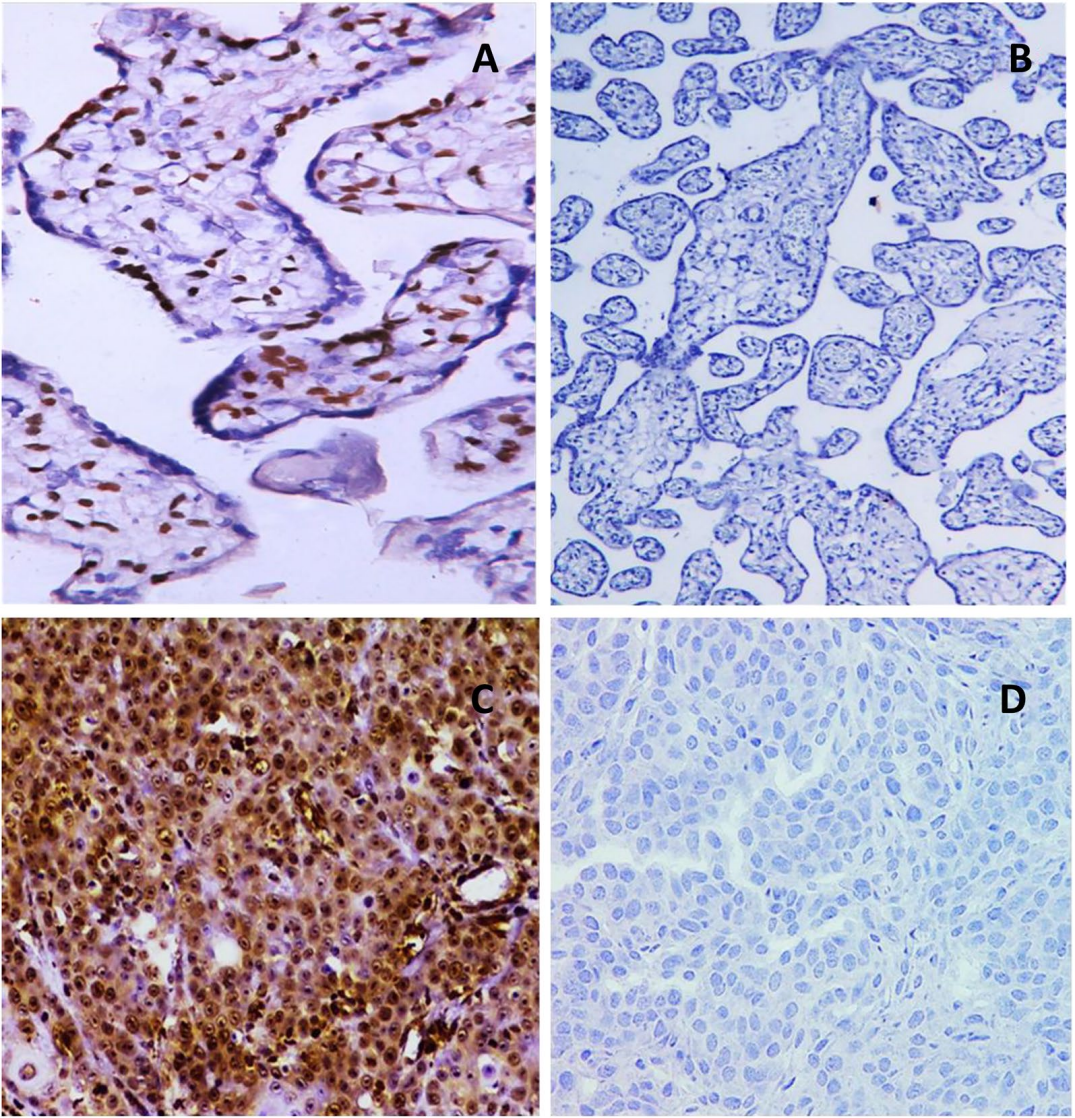
**A** and **B** the positive and negative controls of PROX1 in human placental tissue, **C** and **D** MTA1 positive and negative controls of high-grade breast carcinoma (ABC-DAB, X250, X400)

Another four-micron-thick consecutive sections were cut from the paraffin blocks of the tumors and the control groups for immunostaining. Deparaffinization is followed by rehydration in lowering alcohol grades, followed by water. Antigen retrieval was carried out in a microwave for 10 min using a 0.01 M citric acid buffer (pH = 6.0). The slices were then incubated in a blocking buffer (3% H_2_O_2_) for 5 min before being washed with distilled water. At 1/200 and 1/400 dilutions, anti-PROX1 antibody (Santa Cruz Biotechnology, Inc., Santa Cruz, CA, USA) and anti-MTA1 monoclonal antibody (mouse, Abcam Corporation, ab 84136, UK) were utilized. The immunoreaction was carried out using the streptavidin–biotin complex method, and after an overnight incubation, the tissue slices were analyzed semi-quantitatively, assessing both staining intensity and positivity percentage. The PROX1 immunostaining score was obtained by multiplying the stained area by the intensity by two examiners who were blinded to the clinico-pathological data. The stained area was evaluated from 0 to 3: 0, none, 1, 10%, 2, 10–50%, and 3, > 50%. The intensity of staining was also rated from 0 to 3; 0, no staining of cancer cells, 1, mild staining of cancer cells, 2, moderate staining of cancer cells, and 3, severe staining of cancer cells. Samples with a total score of ≥ 6 were labelled as PROX1-positive, while those with a total score of < 6 were labelled as PROX1-negative [9]. Moreover, the percentage of positively stained cancer cells (0: 0–5%, 1: 6–25%, 2: 26–50%, 3: 51–75%, 4: > 76%) and the staining intensity (0, negative staining; 1, mild staining; 2, moderate staining; 3, intense staining) were assessed using a semi-quantitative scoring system to determine MTA1 positivity. The staining intensity and percentage of positive cells were multiplied to get the total score, which was rated as 0, 1, 2, 3, 4, 6, 8, 9, or 12. High MTA1 expression was defined as a final staining score of greater than 6, and low MTA1 expression as a final staining score of less than 6 [20]. Tissues from human placenta and high-grade breast carcinoma were used as positive and negative controls for PROX1 and MTA1 antibodies, respectively, according to the manufacturer’s instructions (Fig. 1).

### Statistical analysis

The Pearson chi-square test was used to analyze the data. All investigations included two-sided *P*-values. The Kaplan–Meier approach was used to compute and display DFS and OS, which were then analyzed using the log-rank test. To identify the independent prognostic factor, univariate and multivariate survival analyses were performed using the Cox regression model. A *P*-value of 0.05 or less was judged statistically significant. The data were statistically analyzed using the Excel program and the Statistical Package for Social Science (SPSS) version 22 program.

## Results

### The clinicopathological characteristics of the considered cases

**Fig. 2:**
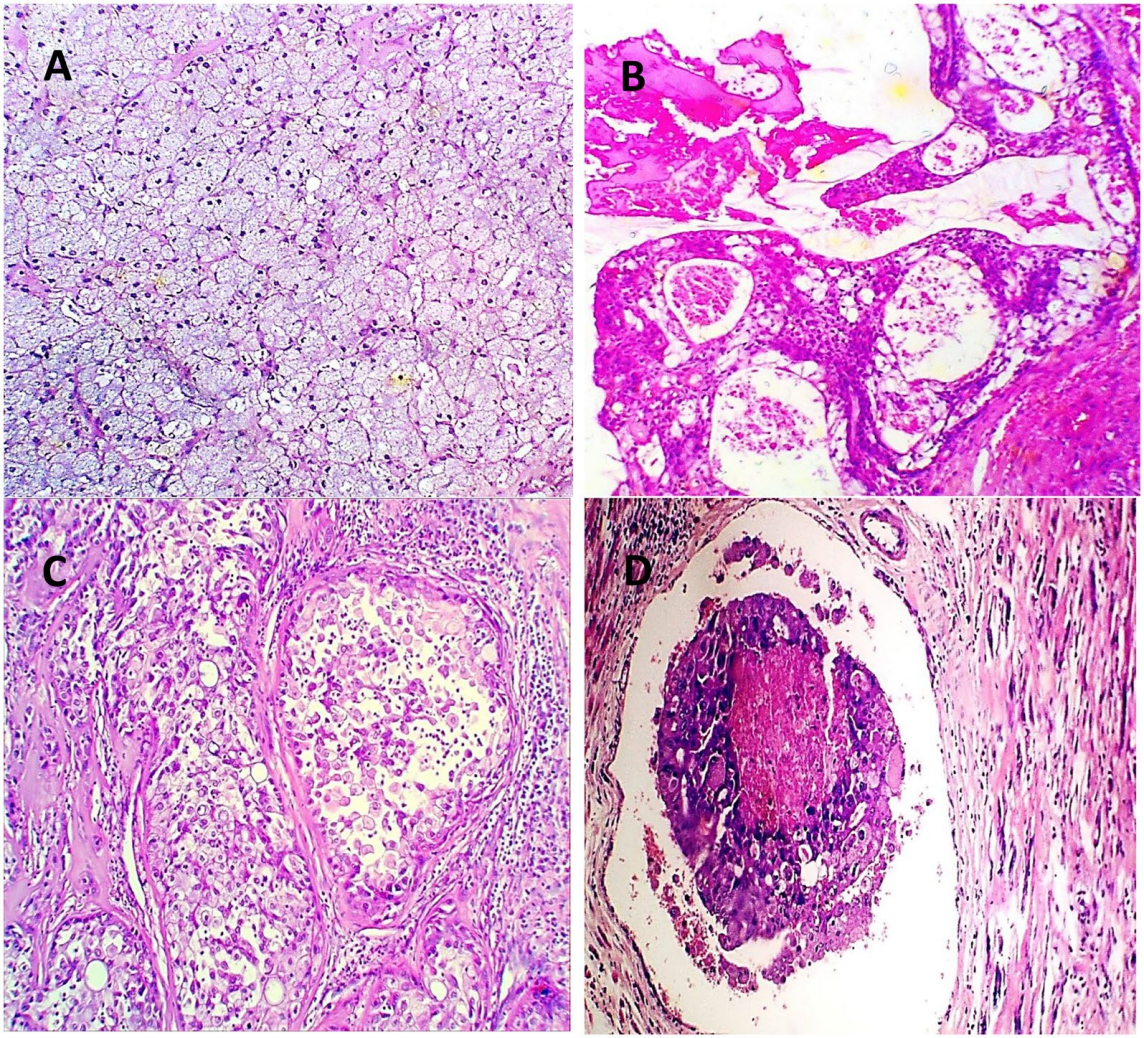
Hematoxylin and eosin-stained sections for **A** Acinic cell carcinoma. **B** low grade MEC, **C** high grade MEC, **D** vascular emboli in high grade MEC (H&E ×400)

**Fig. 3:**
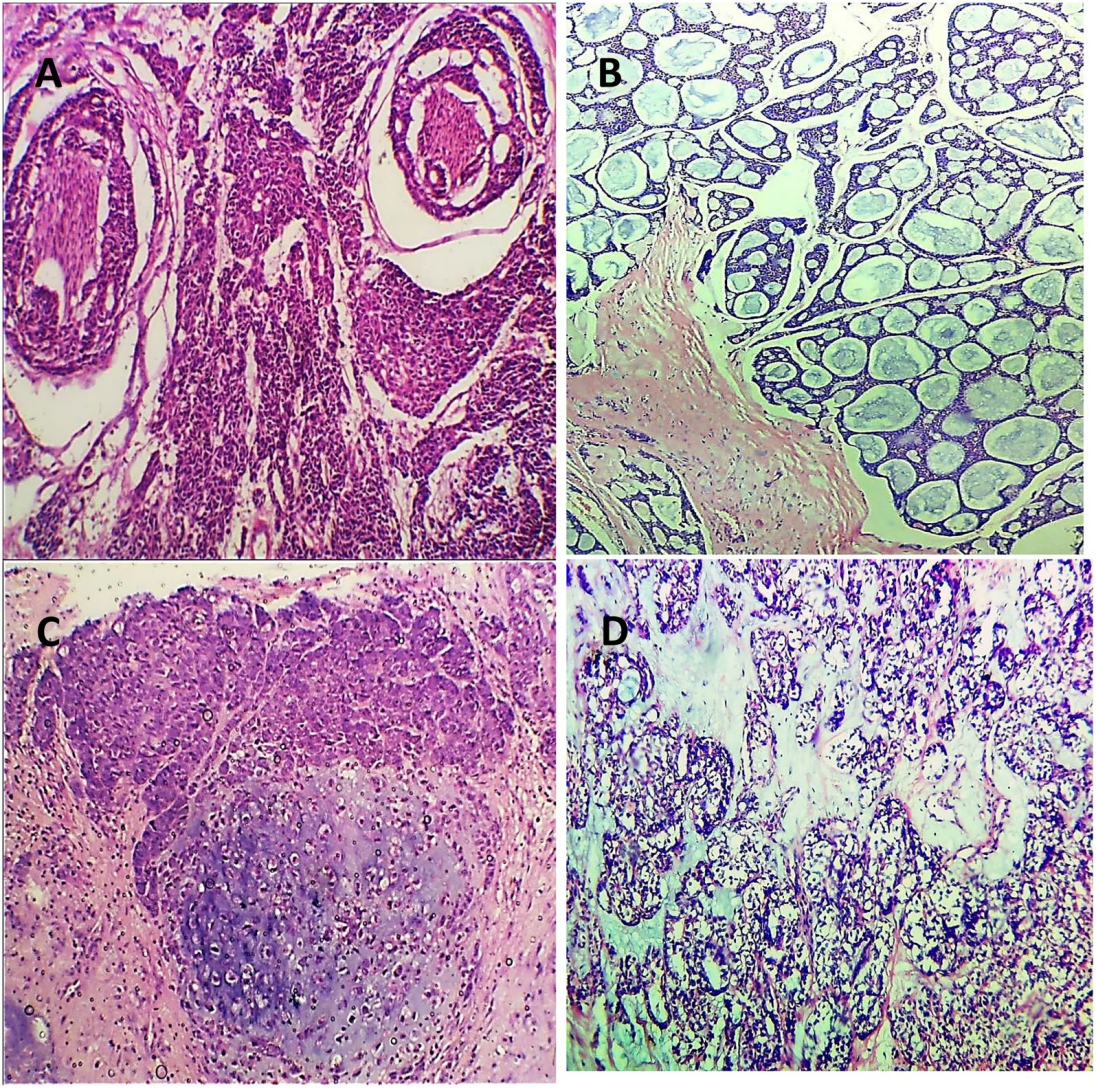
Hematoxylin and eosin-stained sections for **A** Solid and **B** Cribriform pattern of ADCC, **C**, **D** CXPA invading chondroid tissue (H&E ×250)

Sixty percent of the evaluated cases (27 cases) were females, with a female to male ratio of 1.5 to 1. The evaluated cases ranged in age from 35 to 90 years, with a mean of 65.69 years. The working sample included 45 SGCs, with 13 cases of high-grade MEC, 12 cases of AdCC, and 8 cases of CXPA indicating high-grade SGCs, and seven cases of low-grade MEC and five cases of ACC representing low-grade SGCs (Figs. 2, 3). Table 1 shows the clinicopathological characteristics of the worked cases.

### PROX1 immunohistochemical expression concerning the different clinicopathological variables

PROX1 exhibited nuclear and cytoplasmic reactions in tumor cells. About two-thirds of SGCs showed positive PROX1 expression (29 cases, 64.4%), while the remaining one-third presented negative expression (16 cases, 35.6%). Using the Pearson’s Chi-square test, there were no statistically significant variations in PROX1 expression concerning the factors; patient age (*P* = 0.157), gender (*P* = 0.373), tumor site (*P* = 0.801), and incidence of mortality (*P* = 0.078). PROX1 expression, on the other hand, differed significantly by tumor size (*P* = 0.000), incidence of nodal (*P* = 0.007) and distant metastasis (*P* = 0.001), TNM clinical stage (*P* = 0.000), and incidence of tumor recurrence (*P* = 0.001, Table 2).

**Table 2:** PROX1 expression in relation to different clinicopathological parameters

Clinicopathologic variables	PROX1 expression		Total	*P* value/test
	Positive	Negative		
Patients gender				
Male	13 (72.2%)	5 (27.8%)	18 (100%)	0.373/*X*^2^
Female	16 (59.3%)	11 (40.7%)	27 (100%)	
Age group				
< 65 years	10 (52.6%)	9 (47.4%)	19 (100%)	0.157/*X*^2^
≥ 65 years	19 (73.1%)	7 (26.9%)	26 (100%)	
Tumor site				
Parotid salivary gland	15 (65.2%)	8 (34.8%)	23 (100%)	0.801/One-way ANOVA
Submandibular MSG	7 (58.3%)	5 (41.7%)	12 (100%)	
Soft + hard palate minor salivary glands	4 (80%)	1 (20%)	5 (100%)	
Sublingual MSG	2 (50%)	2 (50%)	4 (100%)	
Other sites	1 (100%)	0 (0%)	1 (100%)	
Tumor size groups				
T1 + T2	6 (31.6%)	13 (68.4%)	19 (100%)	0.000/*X*^2^
T3 + T4	23 (88.5%)	3 (11.5%)	26 (100%)	
Nodal involvement				
Positive	21 (80.8%)	5 (19.2%)	26 (100%)	0.007/*X*^2^
Negative	8 (42.1%)	11 (57.9%)	19 (100%)	
Incidence of metastasis				
Present	16 (94.1%)	1 (5.9%)	17 (100%)	0.001/*X*^2^
Absent	13 (46.4%)	15 (53.6%)	28 (100%)	
TNM clinical stage				
Stage I + II	3 (23.1%)	10 (76.9%)	13 (100%)	0.000/*X*^2^
Stage III + IV	26 (81.3%)	6 (18.8%)	32 (100%)	
Tumor type				
Low grade MEC	1 (14.3%)	6 (85.7%)	7 (100%)	0.000/One-way ANOVA
Acinic cell carcinoma	0 (0%)	5 (100%)	5 (100%)	
High grade MEC	10 (76.9%)	3 (23.1%)	13 (100%)	
Adenoid cystic carcinoma	11 (91.7%)	1 (8.3%)	12 (100%)	
Carcinoma ex pleomorphic adenoma	7 (87.5%)	1 (12.5%)	8 (100%)	
Histologic grade				
Low grade carcinomas	1 (8.3%)	11 (91.7%)	12 (100%)	0.000/*X*^2^
High grade carcinomas	28 (84.8%)	5 (15.2%)	33 (100%)	
Lymph vascular invasion				
Present	24 (96%)	1 (4%)	25 (100%)	0.000/*X*^2^
Absent	5 (25%)	15 (75%)	20 (100%)	
Incidence of recurrence				
Present	16 (94.1%)	1 (5.9%)	17 (100%)	0.001/*X*^2^
Absent	13 (46.4%)	15 (53.6%)	28 (100%)	
Incidence of death				
Died	5 (100%)	0 (0%)	5 (100%)	0.078/*X*^2^
Alive	24 (60%)	16 (40.0%)	40 (100%)	

#### *X*^2^ Chi square test, one-way ANOVA test

Pearson’s Chi-square test revealed a highly statistically significant difference in PROX1 immuno-expression between different tumor sizes (*P* = 0.000); with large-sized carcinomas (T3 and T4) showing mainly positive PROX1 expression (23 cases, 88.5%), while the small-sized carcinomas (T1 and T2) showed negative expression (13 cases, 68.4%). In terms of nodal involvement, positive nodal metastasis was identified in 26 cases of the sample studied (57.7%). Significantly, twenty-one of those cases (80.8%) presented a positive PROX1 expression (*P* = 0.007). The cases that were free from nodal metastasis revealed negative (11 cases, 57.9%) and positive (8 cases, 42.1%) PROX1 expression as well. Furthermore, distant metastasis was found in 17 of the SGC cases (37.7%). Positive PROX1 expression was found in a larger number of these patients (16 cases, 94.1%, *P* = 0.001). Pearson’s Chi-square test demonstrated statistically significant differences in PROX1 expression in terms of nodal and distant metastases (*P* = 0.007, 0.001, respectively).

PROX1 expression changed considerably between TNM clinical stages. Cases of advanced clinical stages (III and IV) had carcinomas presented mainly positive PROX1 expression (26 cases, 81.3%). The greater percentage of stage I and stage II cases had carcinomas revealed negative PROX1 expression (10 cases, 76.9%, *P* = 0.000).

Recurrence was reported in 17 of the worked SGC patients (37.7%) during the periodic follow-up events following treatment. Positive PROX1 expression was found in most recurring cases (16 cases, 94.1%). The Pearson`s chi-square test revealed a high statistically significant difference in PROX1 expression in terms of recurrence (*P* = 0.000).

Death occurred in five of the studied cases. All these cases had carcinomas with positive PROX1 expression (100%). Although positive PROX1 expression was found in 60% of the alive patients, the Pearson`s chi-square test revealed no statistically significant difference between PROX1 expression and the risk of mortality (*P* = 0.078).

**Fig. 4:**
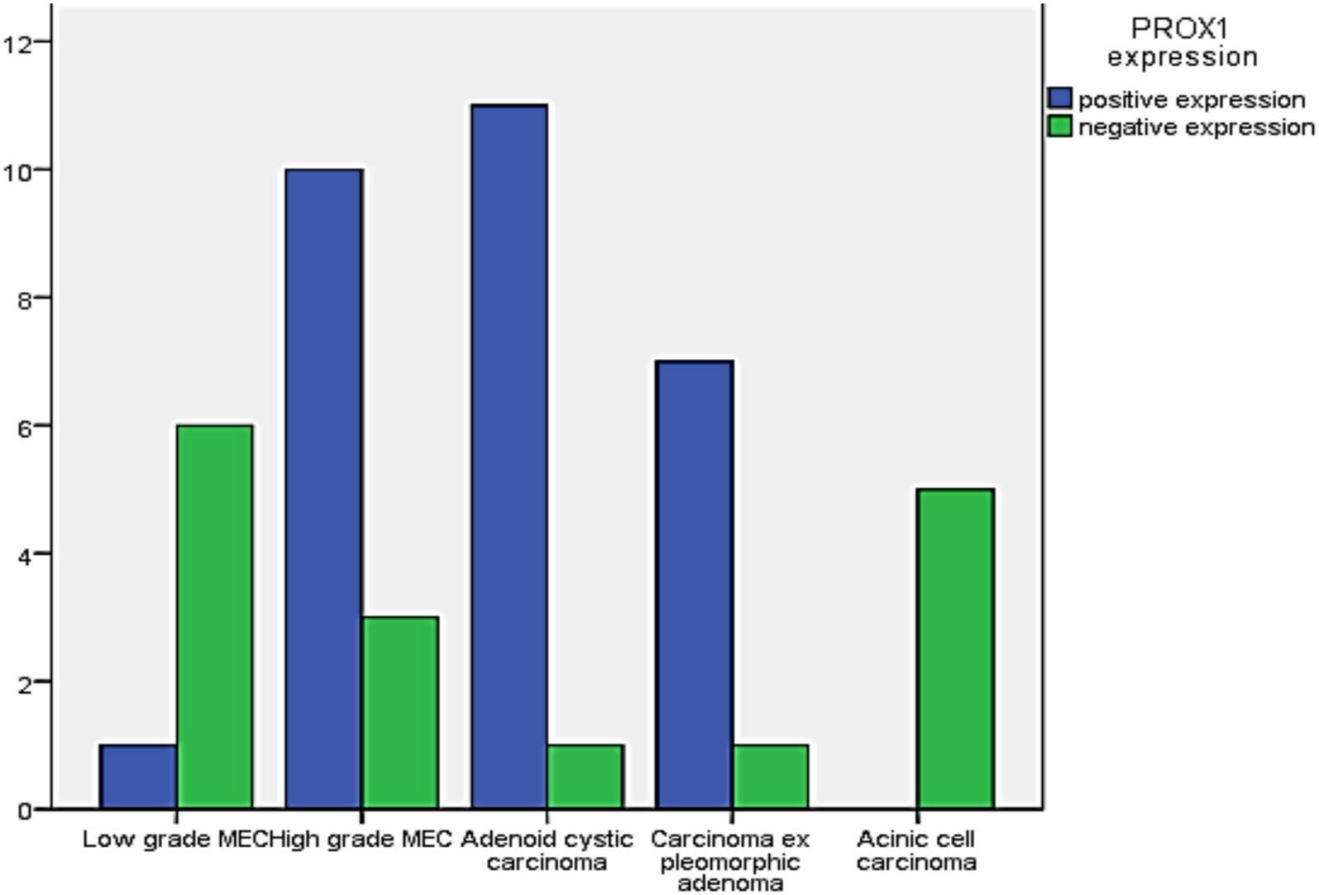
PROX1 immunohistochemical expression concerning the different histologic types of SGC

**Fig. 5:**
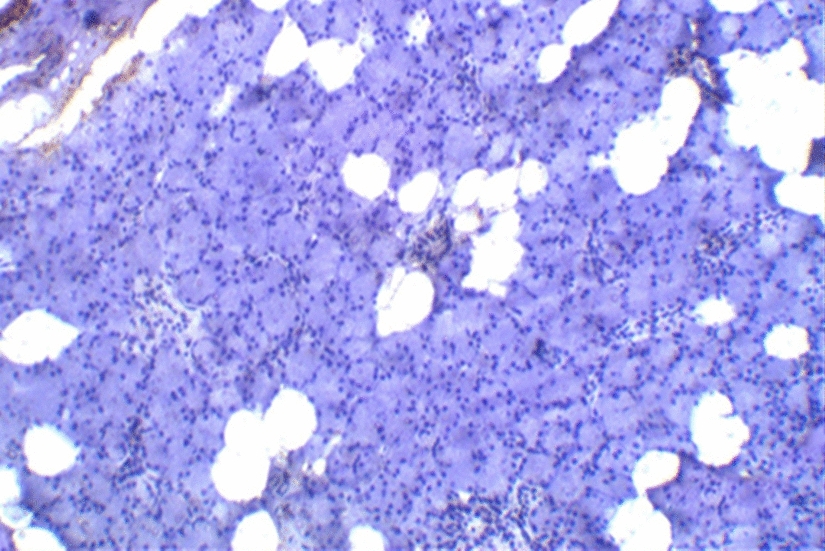
Negative PROX1 expression in normal salivary glands in mucocele ABC-DABx200)

**Fig. 6:**
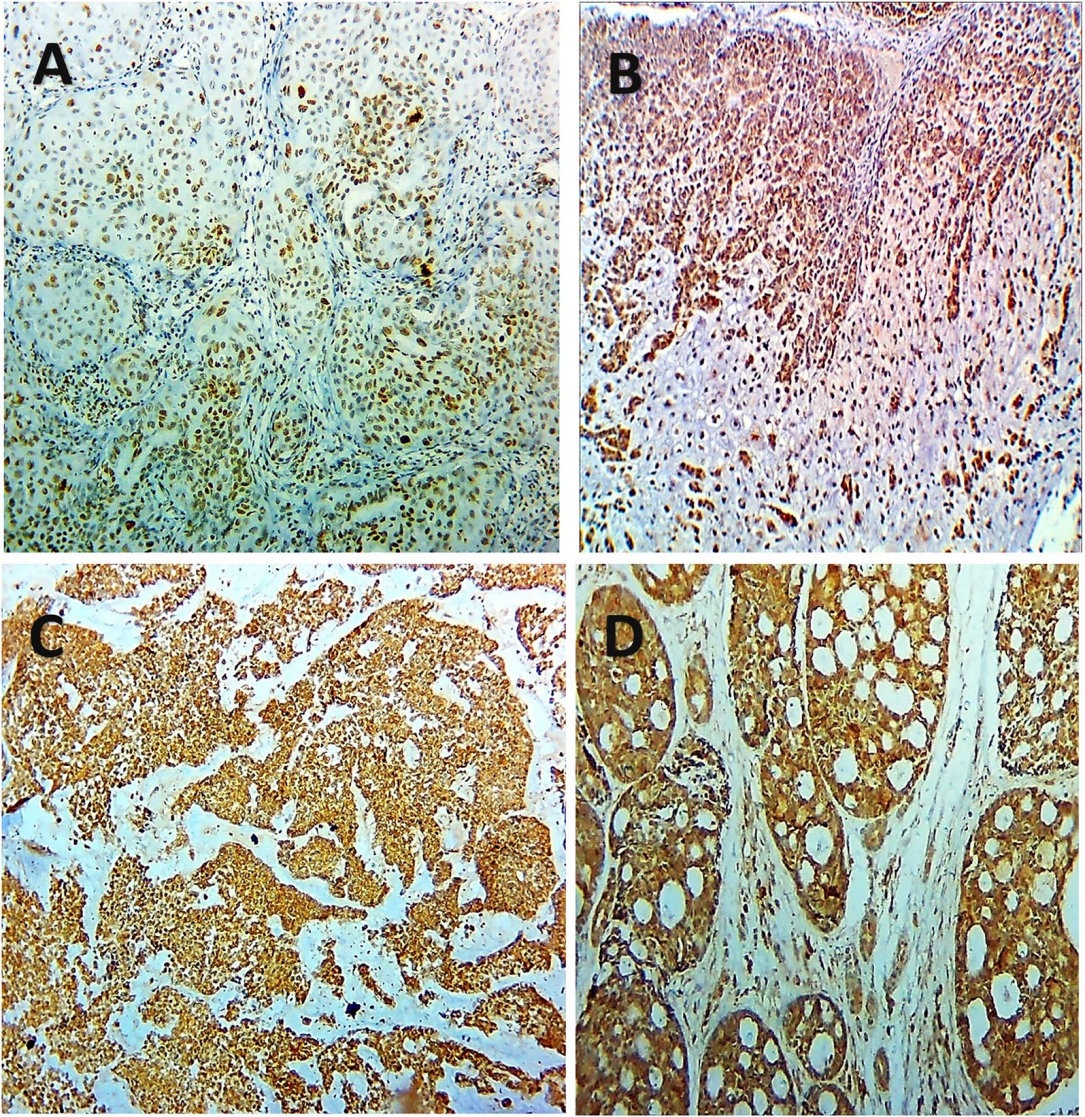
Positive PROX1 expression was observed in high grade MEC (**A**), **B** CXPA, **C** solid and **D** cribriform ADCC (ABC-DABx250)

**Fig. 7:**
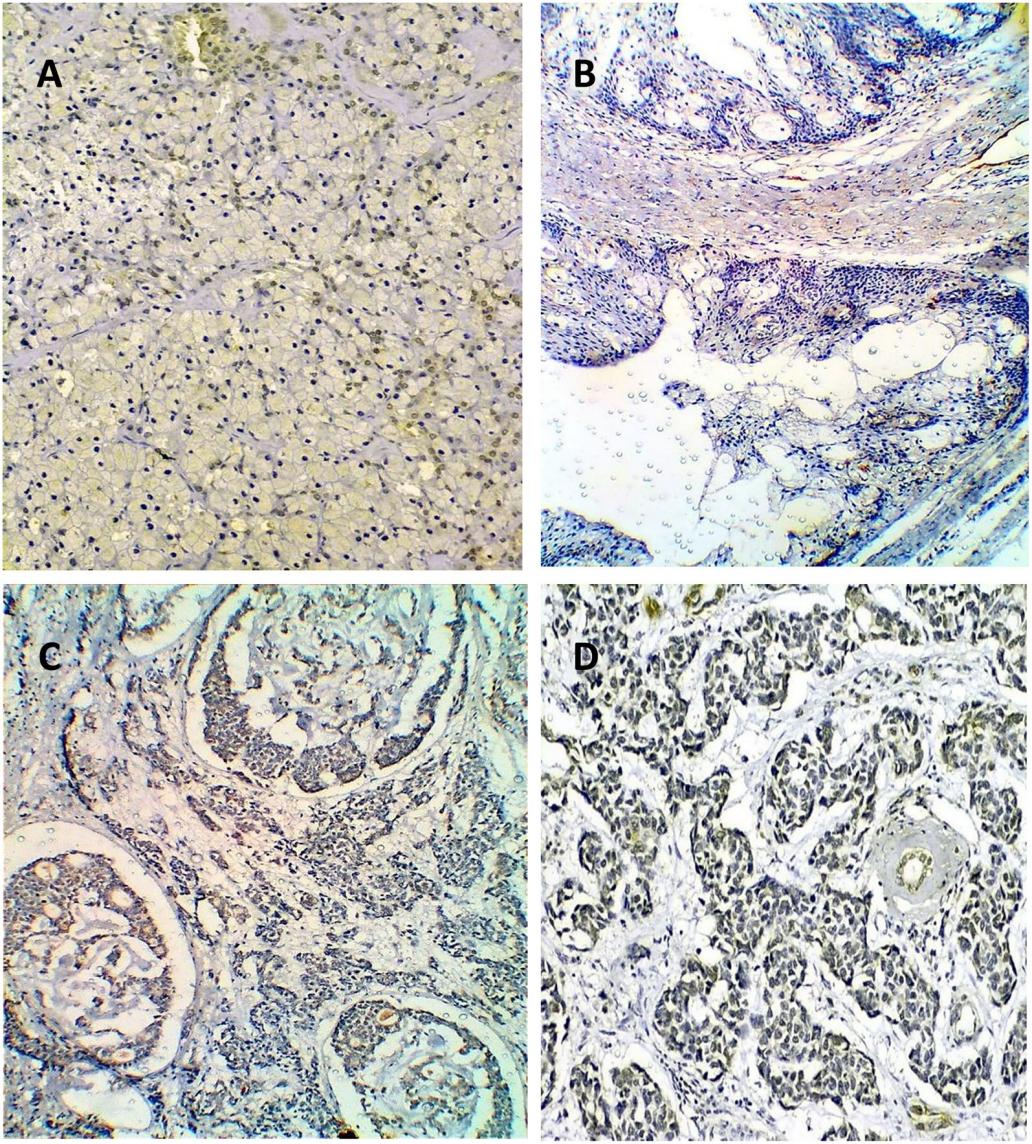
Negative PROX1 expression was noted in (**A**) ACC, **B** low grade MEC, **C** ADCC, and **D** CXPA (ABC-DAB×250, ×400)

In terms of pathological parameters, there were statistically significant differences in PROX1 expression based on the histologic type and grade of SGC, as well as the incidence of lymph vascular invasion (*P* = 0.000). PROX1 expression was mostly positive in high-grade carcinomas (28 instances, 84.8%). Low-grade carcinomas, on the other hand, mostly showed negative expression (11 cases, 91.7%). The majority of AdCC (11 cases, 91.7%), CXPAs (7 cases, 87.5%), and more than three-quarters of high-grade MECs (10 cases, 76.9%) expressed PROX1. Most low-grade carcinomas had negative PROX1 expression, including all five cases of ACC (100%), and six cases of low-grade MEC (85.7%). PROX1 expression was shown to be negative in three cases of high-grade MEC (23.1%), one case of CXPA (12.5%), and one case of AdCC (8.3%, Figs. 4, 5, 6, 7). The existence of lymph vascular tumor emboli was found in 25 (55.6%) of the examined cases. Significantly, positive Prox1 expression was detected in 24 (96%) of those cases. PROX1 was (predominantly) expressed negatively in carcinomas with no lymph-vascular invasion (*P* = 0.000).

### MTA1 immunohistochemical expression concerning the different clinicopathological variables

MTA1 showed a mixed nuclear and cytoplasmic expression throughout the worked SGCs. Of the cases that were examined, more than half had high MTA1 expression (25 cases, 55.6%), low expression was seen in 17 cases (37.8%), and three cases (6.6%) were negative. Using the Pearson’s chi-square test, there were no statistically significant differences in MTA1 expression among the different groups regarding the clinical variables; patient’s age (*P* = 0.213), gender (*P* = 0.176), tumor site (*P* = 0.702), and the incidence of death (*P* = 0.105). However, there were high statistically significant differences in MTA1 expression when it came to the following variables: tumor size (*P* = 0.000), the incidence of nodal (*P* = 0.002) and distant metastasis (*P* = 0.000), TNM clinical stage (*P* = 0.000), and the incidence of tumor recurrence (*P* = 0.000, Table 3).

**Table 3:** MTA1 expression in relation to different clinicopathological parameters

Clinicopathologic variables	MTA1 expression			Total	*P* value/test
	High	low	Negative		
Patients gender					
Male	13 (72.2%)	4 (22.2%)	1 (5.6%)	18 (100%)	0.176/*X*^2^
Female	12 (44.4%)	13 (48.1%)	2 (7.4%)	27 (100%)	
Age group					
< 65 years	8 (42.1%)	10 (52.6%)	1 (5.3%)	19 (100%)	0.213/*X*^2^
≥ 65 years	17 (65.4%)	7 (26.9%)	2 (7.7%)	26 (100%)	
Tumor site					
Parotid MSG	13 (56.5%)	9 (39.1%)	1 (4.3%)	23 (100%)	0.702/Oneway ANOVA
Submandibular MSG	6 (50%)	5 (41.7%)	1 (8.3%)	12 (100%)	
Soft + hard palate minor SG	4 (80%)	1 (20%)	0 (0%)	5 (100%)	
Sublingual MSG	2 (50%)	1 (25%)	1 (25%)	4 (100%)	
Other sites	0 (0%)	1 (100%)	0 (0%)	1 (100%)	
Tumor size groups					
T1 + T2	4 (21.1%)	12 (63.2%)	3 (15.8%)	19 (100%)	0.00/*X*^2^
T3 + T4	21 (80.8%)	5 (19.2%)	0 (0.0%)	26 (100%)	
Nodal involvement					
Positive	20 (76.9%)	6 (23.1%)	0 (0.0%)	26 (100%)	0.002/*X*^2^
Negative	5 (26.3%)	11 (57.9%)	3 (15.8%)	19 (100%)	
Incidence of metastasis					
Present	16 (94.1%)	1 (5.9%)	0 (0.0%)	17 (100%)	0.000/*X*^2^
Absent	9 (32.1%)	16 (57.1%)	3 (10.7%)	28 (100%)	
TNM clinical stage					
Stage I + II	2 (15.4%)	8 (61.5%)	3 (23.1%)	13 (100%)	0.000/*X*^2^
Stage III + IV	23 (71.9%)	9 (28.1%)	0 (0.0%)	32 (100%)	
Tumor type					
Low grade MEC	1 (14.3%)	4 (57.1%)	2 (28.6%)	7 (100%)	0.005/One-way ANOVA
High grade MEC	9 (69.2%)	3 (23.1%)	1 (7.7%)	13 (100%)	
Adenoid cystic carcinoma	10 (83.3%)	2 (16.7%)	0 (0.0%)	12 (100%)	
Carcinoma ex pleomorphic adenoma	5 (62.5%)	3 (37.5%)	0 (0.0%)	8 (100%)	
Acinic cell carcinoma	0 (0.0%)	5 (100%)	0 (0.0%)	5 (100%)	
Histologic grade					
Low grade carcinomas	1 (8.3%)	9 (75%)	2 (16.7%)	12 (100%)	0.001/*X*^2^
High grade carcinomas	24 (72.7%)	8 (24.2%)	1 (3%)	33 (100%)	
Lymph-vascular invasion					
Present	22 (88%)	2 (8%)	1 (4%)	25 (100%)	0.000/*X*^2^
Absent	3 (15%)	15 (75%)	2 (10%)	20 (100%)	
Recurrence					
Present	16 (94.1%)	1 (5.9%)	0 (0%)	17 (100%)	0.000/*X*^2^
Absent	9 (32.1%)	16 (57.1%)	3 (10.7%)	28 (100%)	
Death					
Died	5 (100%)	0 (0%)	0 (0%)	5 (100%)	0.105/*X*^2^
Alive	20 (50%)	17 (42.5%)	3 (7.5%)	40 (100%)	

#### Pearson’s Chi square test, one-way ANOVA test

A highly statistically significant difference in MTA1 IHC expression between tumor sizes was found using the Pearson's chi-square test (*P* = 0.000). Small-sized carcinomas (T1 and T2) exhibited primarily low (12 cases, 63.2%) and negative expression (3 cases, 15.8%) compared to large-sized carcinomas (T3 and T4), which exhibited primarily high immune-expression (21 cases, 80.8%).

In terms of nodal involvement incidence, 26 of the cases under the study (57.7%) had positive nodal metastases. In almost 75.9% of these cases, MTA1 expression was high. Low (11 cases, 57.9%) and negative (3 cases, 15.8%) MTA1 expression was found in nodal metastasis-free cases. Furthermore, it was noted that 17 of the worked patients (37.7%) had distant metastases. In 16 of these cases, there was high MTA1 expression (94.1%). High statistically significant differences in MTA1 expression were found in relation to the status of nodal (*P* = 0.002) and distant metastasis (*P* = 0.000), according to Pearson’s chi-square test.

There was a notable variation in MTA1 expression throughout the various TNM clinical stages. Most cases (23 cases, 71.9%) in stage III and IV showed significant high MTA1 expression. Conversely, the higher proportion of stage I and stage II patients had negative (3 cases, 23.1%) and low expression (8 cases, 61.5%, *P* = 0.000).

Recurrence was reported in 17 of the handled cases (37.7%) during the periodic follow-up events after the treatment. In sixteen cases, or 94.1%, there was a significant observation of high MTA1 expression. Low (16 cases, 57.1%), high (9 cases, 32.1%), and negative MTA1 expression (3 cases, 10.7%) were exhibited by cases with a negative history of recurrence. When taking the incidence of recurrence into account, Pearson's chi-square test showed a high statistically significant difference in MTA1 expression (*P* = 0.000).

Five of the studied cases had reports of death. These five cases showed high MTA1 expression. Of the 40 individuals who were still alive, 50% (20 cases) had high MTA1 expression; the other 20 cases showed low (17 cases, 42.5%) and negative expression (3 cases, 7.5%). Pearson chi-square test revealed no statistically significant difference in MTA1 expression concerning the incidence of death (*P* = 0.105).

**Fig. 8:**
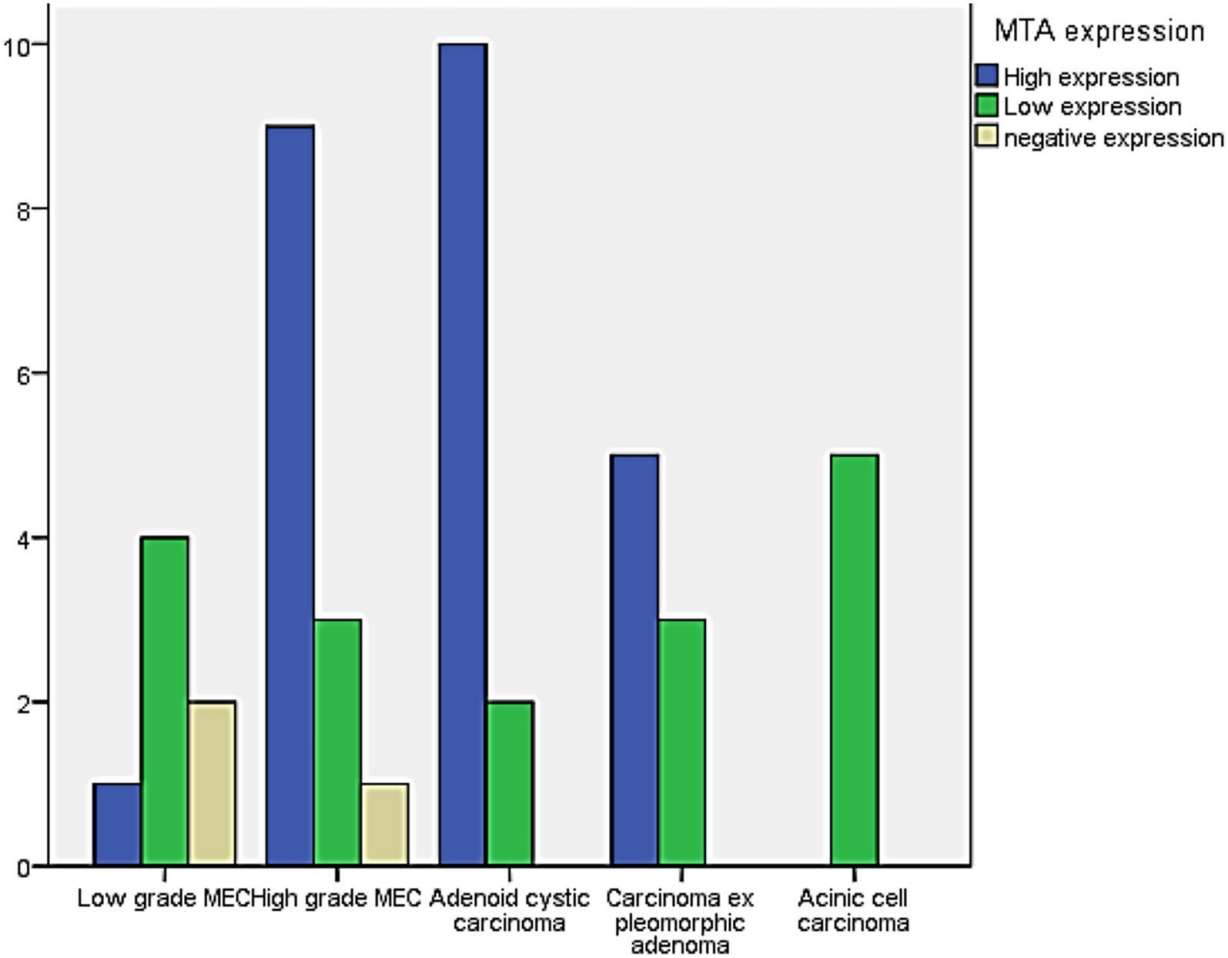
MTA1 immunohistochemical expression concerning the different histologic types of SGC

**Fig. 9:**
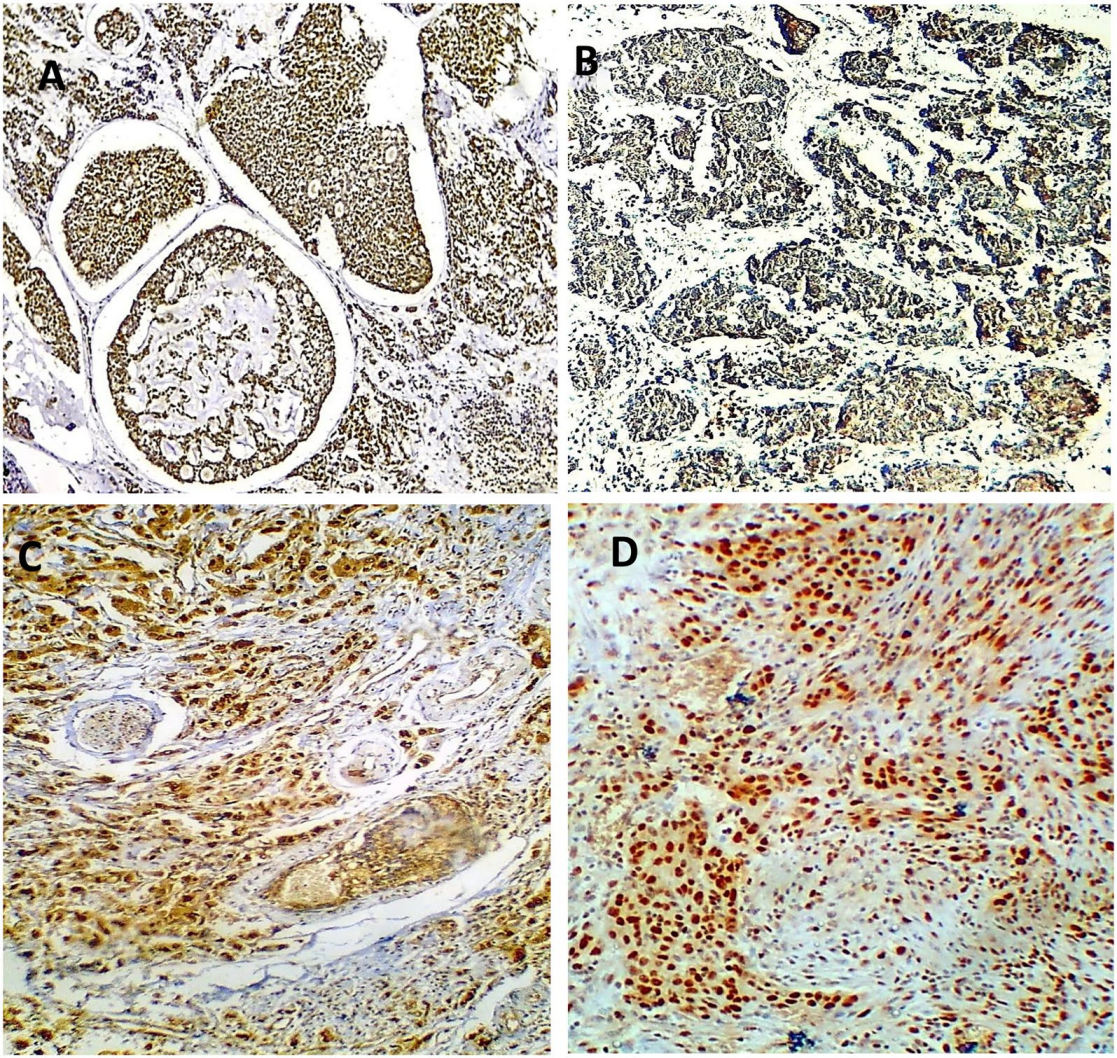
High MTA 1 espression in **A** cribriform and **B** solid ADCC, **C** high grade MEC (perineural invasion), and **D** CXPA (ABC-DAB×250, ×400)

**Fig. 10:**
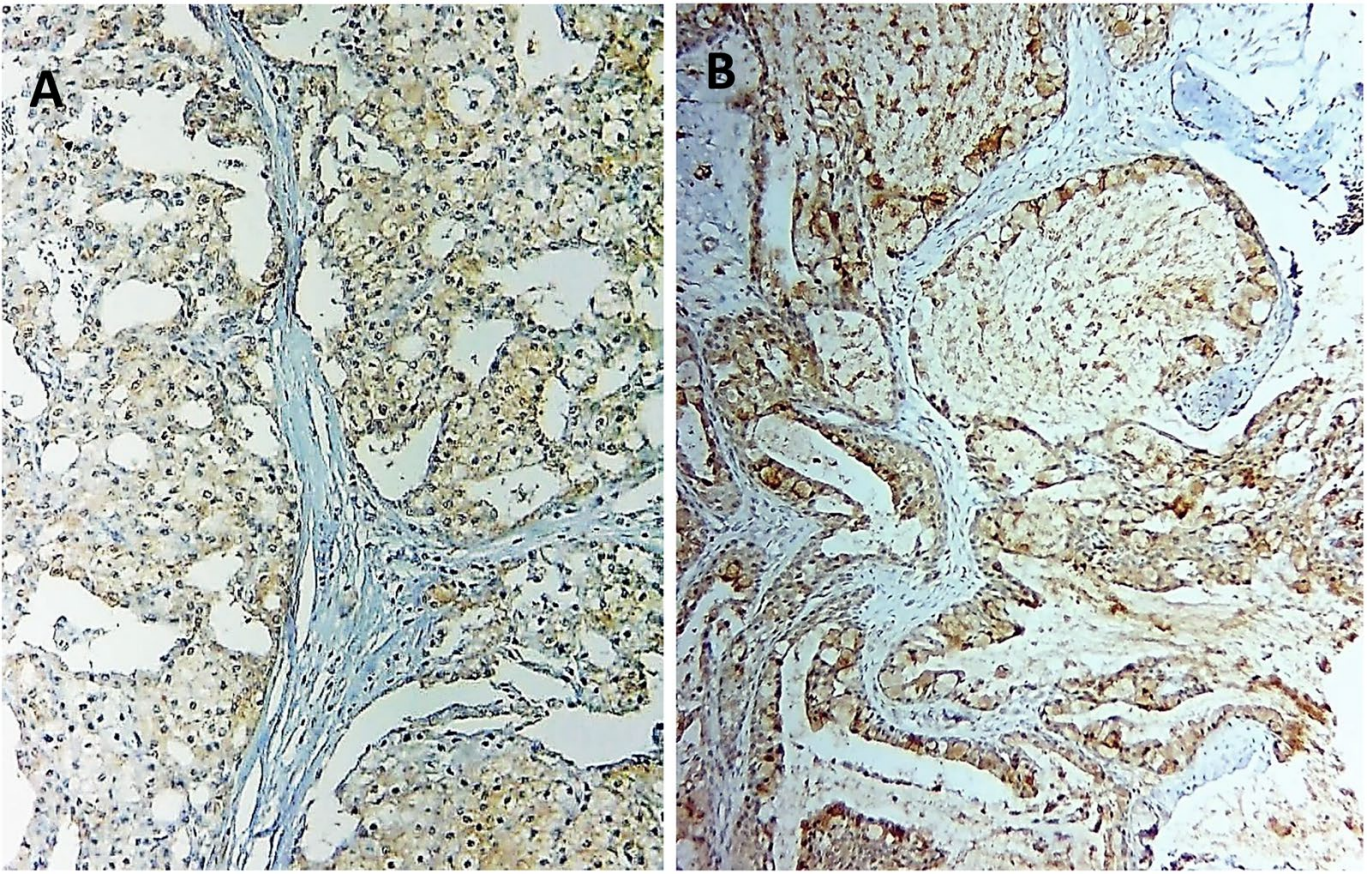
Low MTA1 expression in **A** ACC (papillary cystic type) and **B** low-grade MEC (ABC-DAB ×400)

As regards the pathological variables, there were high statistically significant differences in MTA1 expression considering the following variables: the histological type (*P* = 0.005) and grade of carcinoma (*P* = 0.001), and the incidence of lymph vascular invasion (*P* = 0.000). High-grade tumors revealed mainly high MTA1 expression (24 cases, 72.7%), and about one-quarter of that cases revealed low MTA1 expression (8 cases, 24.2%). On the other hand, low-grade carcinomas mainly demonstrated low (9 cases, 75%), and negative (2 cases, 16.7%) MTA1 expression. High MTA1 expression was observed in 10 cases (83.3%) of ADCCs, nine cases (69.2%) of high-grade MECs, and five cases (62.5%) of CXPAs. Low-grade carcinomas mainly present low and negative MTA1 expression. All five cases (100%) of ACC demonstrated low MTA1 expression, while low-grade MEC revealed low (4 cases, 57.1%) and negative expression (2 cases, 28.6%, Figs. 8, 9, 10).

### The correlation between PROX1 and MTA1 expressions

Relatively, both markers demonstrated similar findings across the worked SGC cases concerning the different clinicopathologic variables. The greater percentage of cases that had positive PROX1 expression revealed high MTA1 expression (25 cases, 86.2%). Moreover, PROX1 negative cases demonstrated low (13 cases, 81.3%), and negative (3 cases, 18.8%) MTA1 expression. Pearson’s and Spearman`s correlation tests presented a strong positive correlation between PROX1 and MTA1 immuno-expression across the studied SGC cases (Pearson’s R = 0.812, Table 4, 5).

**Table 4:** The correlation between PROX1 and MTA1 immunohistochemical expressions in the worked SGC cases

	MTA1 expression			Total
	High	Low	Negative	
PROX1 expression				
Positive				
Count	25	4	0	29
% within PROX1 expression	86.2%	13.8%	0.0%	100.0%
Negative				
Count	0	13	3	16
% within PROX1 expression	0.0%	81.3%	18.8%	100.0%
Total				
Count	25	17	3	45
% within PROX1 expression	55.6%	37.8%	6.7%	100.0%

**Table 5:** The correlation between PROX1 and MTA1 expressions in SGC cases

		Value	Asymp. std. error^a^	Approx. *T*^b^	Approx. sig
Nominal by nominal	Contingency Coefficient	0.643			0.000
Interval by interval	Pearson’s *R*	0.812	0.050	9.110	0.000
Ordinal by ordinal	Spearman correlation	0.837	0.063	10.015	0.000
No. of valid cases		45			

### Disease free survival (DFS) and overall survival (OS)

**Fig. 11:**
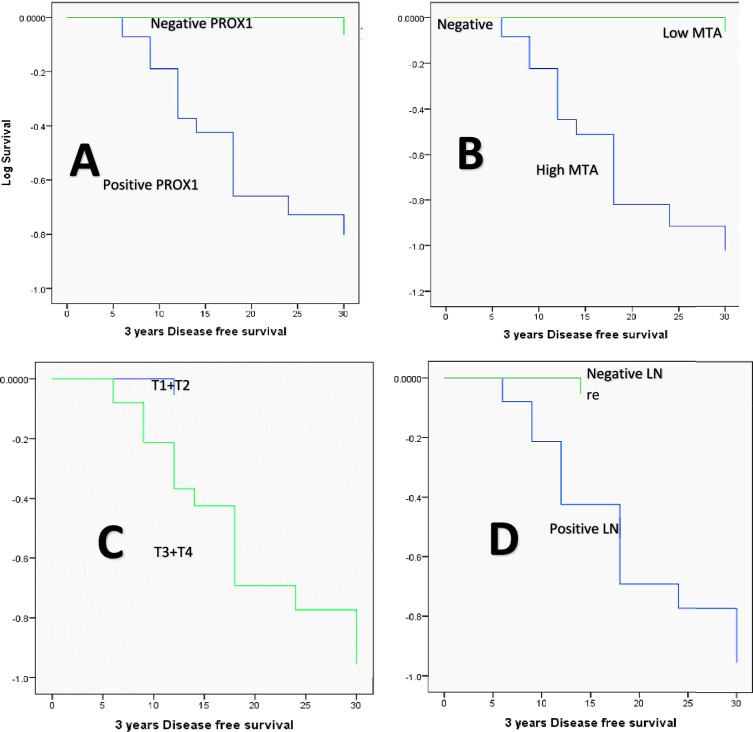

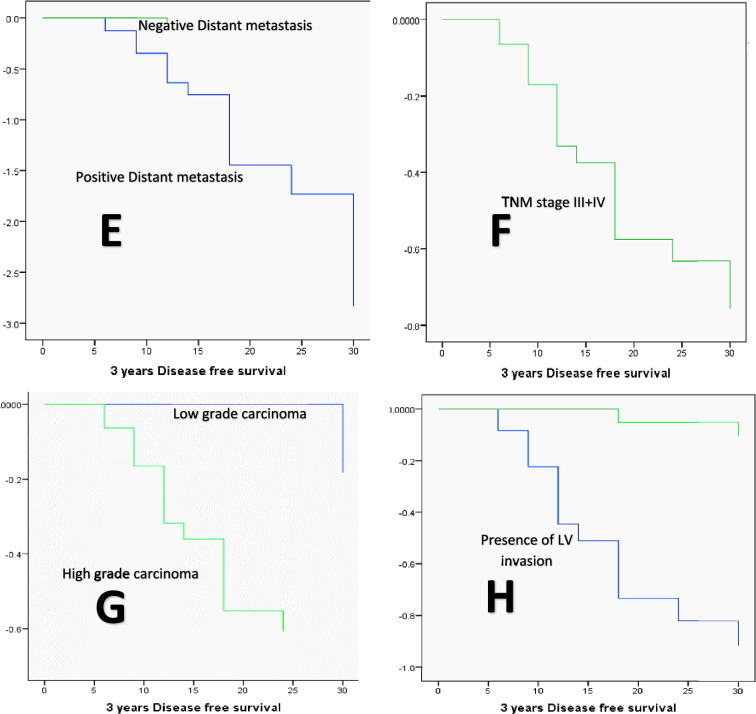
The Kaplan Meier survival plots demonstrate that DFS was significantly reduced concerning (**A**) positive PROX1 expression, **B** high MTA1 expression, **C** T3 + T4 tumor size group, **D** Positive nodal involvement, **E** presence of distant metastasis, **F** advanced TNM clinical stage (III + IV), **G** high grade carcinomas, and **H** presence of LV invasion

**Fig. 12:**
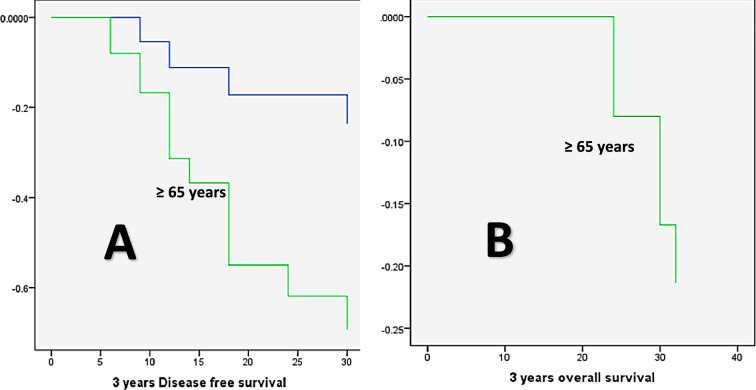
Kaplan–Meier survival plots demonstrate (**A**) DFS, **B** OS were significantly reduced in old age patients (≥ 65 years)

The Kaplan–Meier method, the log-rank test, and the Cox regression model were used to analyze the patients’ DFS and OS in relation to the various clinicopathologic factors. DFS was significantly lower in cases with positive PROX1 expression (23.966 months versus 35.625 months for negative PROX1 expression, *P* = 0.001), high MTA1 expression (22.04 months versus 35.64 and 36 months for low and negative MTA1 expression, respectively, *P* = 0.000), large-sized tumors (T3 + T4, 23.269 months versus 34.737 months for small-sized tumors, *P* = 0.000), and positive nodal involvement (23.192 months versus 34.84 months for negative nodal involvement cases, *P* = 0.000), large-sized tumors (T3 + T4, 23.269 months versus 34.737 months for small-sized tumors, *P* = 0.000), Advanced TNM clinical stage (III + IV, 24.906 months versus 36 months for stage I and II cases, *P* = 0.002), high-grade carcinomas (25.606 months versus 35 months for low grade carcinoma, *P* = 0.05), the presence of LVI (22.76 months versus 34.8 months, *P* = 0.000), old age (≥ 65 years, 25.231 months versus 32.053 months for age group younger than 65 years, *P* = 0.047), and positive distant metastasis (16.529 months versus 35.143 months for negative cases, *P* = 0.000). On the other hand, DFS did not show a statistically significant difference concerning the tumor site factor (*P* = 0.419) or gender (*P* = 0.505, Figs. 11, 12). Distant metastasis was identified as the independent predictor of DFS by multivariate analysis utilizing the Cox regression model (*P* = 0.000, Table 6).

**Table 6:** The Cox regression model illustrates the independent predictor(s) of DFS

Variables	*B*	SE	Wald	d*f*	Sig	Exp(*B*)	95.0% CI for Exp(*B*)	
							Lower	Upper
Distant metastasis	1.794	0.445	16.274	1	0.000	6.016	2.516	14.386
Histologic grade	− 0.081	0.471	0.030	1	0.863	0.922	0.366	2.319
PROX1	− 0.051	0.662	0.006	1	0.939	0.950	0.259	3.482
MTA1			0.046	2	0.977			
MTA1 (1)	0.100	0.900	0.012	1	0.911	1.105	0.190	6.446
MTA1 (2)	− 0.024	0.670	0.001	1	0.971	0.976	0.262	3.631
TNM stage	− 0.029	0.431	0.004	1	0.947	0.972	0.418	2.259

**Fig. 13:**
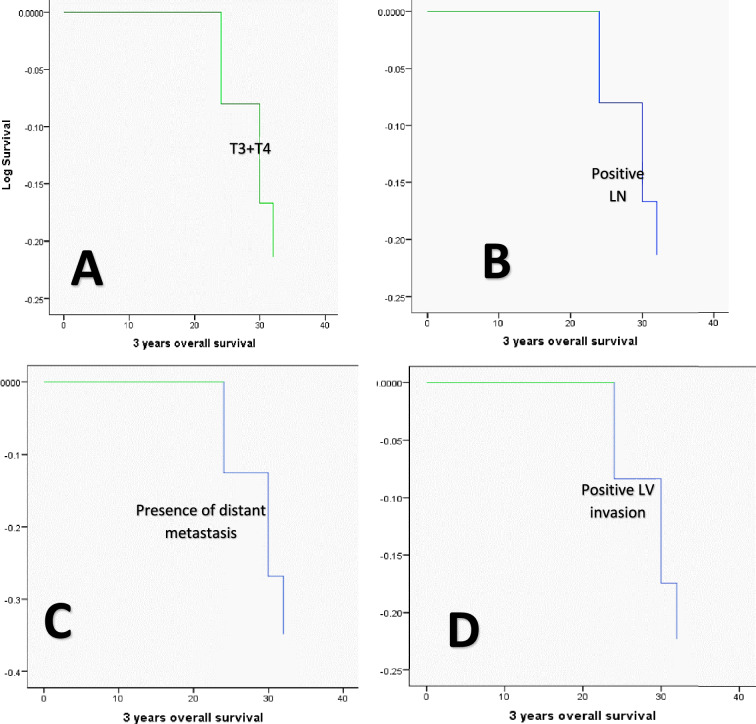

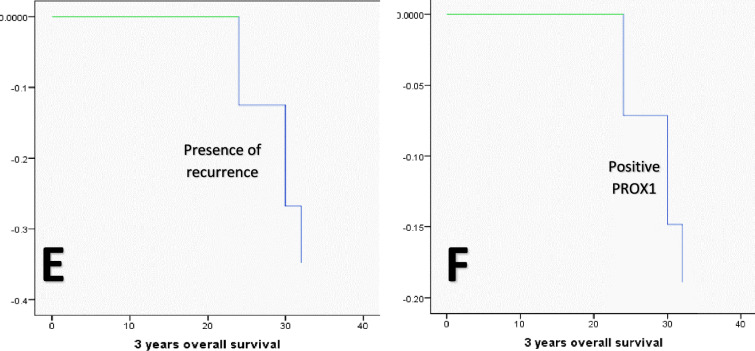
Kaplan Meier survival plots demonstrate that OS was significantly reduced concerning (**A**)T3 + T4 tumor size group, **B** presence of nodal involvement, **C** presence of metastasis, **D** Presence of LV invasion, **E** presence of recurrence, and **F** positive PROX1 expression

Using the Kaplan Meier method, the univariate analysis of OS concerning the various clinicopathological variables showed that OS was significantly lower in cases with large-sized tumors (T3 + T4, 28 months versus 36 months for T1 and T2 tumors, *P* = 0.046), positive nodal involvement (30 months versus 36 months for negative nodal involvement, *P* = 0.046), positive distant metastasis (33.647 months versus 36 months for negative distant metastasis cases, *P* = 0.002), presence of recurrence during follow-up (31.23 months versus 36 months for recurrence free cases, *P* = 0.002), positive PROX1 expression (29.43 months versus 35.45 months for negative PROX1 expression cases, *P* = 0.05), presence of LVI (33 months versus 36 months for absence of LVI cases, *P* = 0.037), and old age group (≥ 65 years, 34.46). However, when it came to MTA1 expression (*P* = 0.113), tumor grade (*P* = 0.162), tumor histologic type (0.091), TNM stage (*P* = 0.140), tumor site (*P* = 0.789), and gender variables (*P* = 0.059), there were no statistically significant differences observed in OS. None of the variables under investigation were able to predict the OS, according to the multivariate analysis conducted using the Cox regression model (Table 7 and Fig. 13).

**Table 7:** The Cox regression model illustrates the independent predictor(s) of OS

Variables	*B*	SE	Wald	d*f*	Sig	Exp(*B*)	95.0% CI for Exp(*B*)	
							Lower	Upper
Distant metastasis	0.313	0.404	0.600	1	0.439	1.367	0.620	3.015
Histological grade	− 0.107	0.466	0.053	1	0.819	0.899	0.360	2.241
PROX1	− 0.039	0.662	0.003	1	0.953	0.962	0.263	3.518
MTA1			0.003	2	0.999			
MTA1 (1)	0.018	0.901	0.000	1	0.984	1.018	0.174	5.950
MTA1 (2)	− 0.012	0.670	0.000	1	0.986	0.988	0.266	3.672
TNM stage	0.025	0.433	0.003	1	0.953	1.026	0.439	2.395

## Discussion

Salivary gland tumors represent an important part of oral and maxillofacial pathology [19]. Malignant salivary gland tumors are rare with variable histological patterns and biological behaviors, so it is mandatory to find reliable biomarkers [6, 8]. These cancer biomarkers play a critical role in oncology, including risk assessment, screening, differential diagnosis, prediction of the prognosis and response to treatment, and monitoring of the disease progression [21].

PROX1 is a fork head-box transcription factor. The protein encoded by the PROX1 gene is a member of the homeobox transcription factor family. It acts as a key regulator in early embryonic development and is involved in lymphatic vessel development [22]. PROX1 has both tumor-suppressive and oncogenic features according to the type of cancer and its expression is changed in a variety of human cancers [8, 23]. Although it has been established that PROX1 is important in the development and metastasis of cancer, it is mostly unknown how it regulates the ability of cancer cells to proliferate, migrate, and invade [22, 24].

In the current study, PROX1 showed both nuclear and cytoplasmic reactions in tumor cells. The cytoplasmic expression of PROX1 is a very controversial issue. Some studies reported PROX1 cytoplasmic expression by IHC staining in different tumor types, as in the case of colorectal cancer. The same study admits that there is a reason for the cytoplasmic localization of the expression because PROX1 is enriched and activated in the cytoplasm of the cell before being translocated to the nucleus to become functionally active [25]. Also, it has been proven that prospero, the Drosophila counterpart of PROX1, is usually found in young proliferating cells’ cytoplasm [26]. Another study in gastric cancer showed both nuclear and cytoplasmic reactions [22].

In contrast to low-grade carcinomas, we observed positive IHC expression of PROX1 in high-grade salivary gland carcinomas. There were significant statistical differences found between the expression of PROX1 and the histological type and grade of carcinoma as well as the incidence of lymph vascular invasion (*P* < 0.000). Additionally, concerning the clinical factors there were significant statistical differences in PROX1 expression based on the following variables: TNM clinical stage, tumor size, nodal and distant metastasis incidence, and tumor recurrence incidence (*P* < 0.05).

Our findings were consistent with other research, as Gao et al. had shown that patients with salivary adenoid cystic carcinoma (SACC) had a lower survival rate and unfavorable clinicopathological characteristics when the high expression of PROX1 was present. It was also discovered that in SACC, there is a correlation between high expression of PROX1 and aggressive oncogenic behavior such as perineural invasion, local regional recurrence, and distant metastasis [8]. Upregulated PROX1 expression was significantly correlated with tumor differentiation, tumor size, metastasis, depth of invasion, and cancer stages in several human tumors, which include gastric cancer [11], hepatocellular carcinoma [27], esophageal squamous cell carcinoma [28], renal cell carcinoma [29]. Another research supported the involvement of PROX1 as one of the main promoters of tumor invasion and metastasis in gastric cancer and suggested its role as a prognostic factor for such malignancies [22].

Additionally, a different research team evaluated the predictive significance of PROX1’s IHC expression in 517 cases of colorectal cancer. They reported that a low degree of tumor differentiation (*P* < 0.0001) was linked to high expression of PROX1 [25]. PROX1 was commonly up-regulated and functioned like oncogene by causing epithelial-mesenchymal transition (EMT) in vitro and in vivo, Zhu et al. found that the WNT signaling pathway may be promoted by elevated PROX1 expression. WNT activation produces a number of integrated EMT-related signals, binds to several receptor types, initiates a range of signal transduction pathways, and promotes cell proliferation. Also, it was discovered that PROX1 facilitated cancer spread via triggering WNT signaling to preserve β-catenin stability and encourage nuclear translocation of β-catenin. Therefore, it was concluded that PROX1 could serve as a substantial therapeutic target as well as an important diagnostic marker for patients with breast cancer [7].

Lu et al. [30] illustrated the relationship between PROX1 and EMT as PROX1 overexpression led to E-cadherin and integrins downregulation and reduction of cell adhesion. These cells exhibited increased invasiveness and matrix metalloproteinase activity. On the other hand, PROX1 knockdown decreased invasiveness and restored E-cadherin protein expression in SW620 cells. Surprisingly, transcriptional inhibition was not the mechanism by which PROX1 repressed E-cadherin. It was discovered that PROX1 suppressed E-cadherin 3′UTR reporter activity and protein expression via binding to the miR-9-2 promoter and inducing its expression. While precursor miR-9 suppressed E-cadherin in PROX1-knockdown cells, anti-miR-9 restored E-cadherin in SW620 cells. Compared to tumor tissues with low PROX1/high E-cadherin, those with high PROX1/low E-cadherin had a higher level of miR-9.

To our knowledge, there is a lack of published studies illustrating the molecular mechanisms underlying the association between PROX1 overexpression and EMT in salivary gland carcinomas. Although PROX1 has been implicated in EMT regulation in other malignancies, its specific mechanistic role in SGC remains unclear.

PROX1 aids in the growth and lymphatic dissemination of neuroblastomas, and there is a correlation between tumor lymphatic density and lymph node metastases [9, 22, 31]. The fact that PROX1 controls the expression of several lymphatic transcription factors, including vascular endothelial growth factors (VEGFs) and the lymphatic vessel endothelial hyaluronan receptor-1 (LYVE-1), demonstrates the correlations between PROX1 expression and lymphovascular invasion, tumor invasion, and metastasis. It’s interesting to note that PROX1 is believed to function as an extra angiogenic factor, primarily by activating VEGF receptor-3 (VEGFR-3). There is an embryogenic ancestor shared by the vascular, lymphatic, and blood systems. PROX1 is one of the many variables that have a significant impact on lymphatic endothelial cell differentiation during development. Interestingly, overexpressing PROX1 can turn blood endothelial cells into lymphatic endothelial cells. Furthermore, the cell identity of the lymphatic endothelium system is variable and highly dependent on PROX1 activity. Accordingly, it’s possible for lymphatic endothelial cells to reversely differentiate into blood endothelial cells in situations when PROX1 is silenced [32, 33].

On the other hand, another study in oral squamous cell carcinoma (OSCC) showed that Local tumor progression, clinical stage, lymphovessel density (LVD), nodal metastasis, and a poorer prognosis were all substantially correlated with Prox1 expression showing that Prox1 activates the expression of vascular endothelial growth factor (VEGF)-C, which controls cell growth, proliferation, lymphangiogenesis and invasion [12, 29]. In contrast, Benitha et al., showed that there was a noteworthy inverse relationship between PROX-1 and histopathological markers, including tumor staging, perineural invasion, lymphovascular invasion, and staging. The PROX-1 and histologically tumor-free margins in patients with disease-free survival showed a strong favorable association [34]. Also, In OSCC cell lines, Rodrigues et al. have shown that PROX-1 expression is downregulated [35]. These differences might be illustrated by that PROX1 has a tumor suppressor gene or an oncogene function in different cancer types [11].

MTA1 is a coregulatory factor that impacts chromosomal remodeling and cellular signaling in addition to PROX1. It contributes to the growth, invasion, and spread of metastatic epithelial cells because it exhibits transcriptional activity [17, 18]. MTA1 overexpression has been observed in several human cancers, and it has been connected to tumor invasion, a higher risk of metastasis, and an advanced clinical stage [36].

High-grade tumors in our investigation primarily showed elevated MTA1 expression. Conversely, low-grade carcinomas primarily showed negative and low expression. There were no statistically significant variations in MTA1 expression across the groups with respect to the clinical characteristics, patient age, gender, tumor site, and death incidence. However, when it came to the following factors: tumor size, incidence of nodal and distant metastases, TNM clinical stage, and incidence of tumor recurrence, there were a high statistically significant difference in MTA1 expression (*P* < 0.000). As regards the pathological variables, there were high statistically significant differences between MTA1 expressions and the following variables: the histological type and grade of carcinoma, and the incidence of lymph vascular invasion (*P* < 0.000).

Our results were in accordance with other studies where Ahmed et al. reported there was no statistically significant correlation between MTA1 level of expression and the clinical parameters, such as patient age, gender, and tumor site in mucoepidermoid carcinoma cases [15]. This finding agreed with other previous studies [37, 38]. Additionally, Ahmed et al. detected a strong correlation between MTA1 expression and the clinical stage and lymph node metastases [15].

These results were consistent with other studies that showed MTA1 protein expression is substantially connected with aggressive tumor growth and positive nodal status in head and neck cancer, according to immunohistochemical analysis in squamous cell carcinoma and nasopharyngeal carcinoma [39, 40]. In addition to that, Lin et al. found that female gender and lymph node metastases were substantially correlated with high MTA1 expression. Multivariate analysis revealed that individuals with OSCC who had higher MTA1 expression had a lower mean survival rate [41]. Song et al., showed that overexpression of MTA1 induced EMT and encouraged OSCC invasion, migration [42].

Additionally, it was found that MTA1 overexpression induces morphological changes from epithelial to mesenchymal. MTA1 knockdown reversed these effects. It is generally known that EMT, which enables epithelial cells to acquire invasive mesenchymal characters. Cells undergoing EMT increase the expression of mesenchymal markers and decrease the expression of the epithelial maker [36, 42]. So, MTA1 reportedly promotes cancer cell invasion and metastasis through EMT [43, 44]. Also, it was discovered that overexpression of MTA1 enhanced the levels of Vimentin and Snail protein and decreased the E-cadherin protein level in vitro and in vivo [42].

In nasopharyngeal cancer, MTA1-mediated metastatic promotion was mediated via hedgehog (Hh) signaling [45] which influences various types of tumors [46, 47]. Numerous elements of Hh signaling have been linked in studies to cancer cell invasion, metastasis, and EMT [48, 49].

Another theory stated that hypoxia-induced factor-1α (HIF-1α) is linked to MTA1 function. MTA1 contributes to tumor angiogenesis by deacetylating HIF-1α and upregulating the production of histone deacetylase-1 [17, 50]. Additionally, MTA1 can bind to HIF-1α and deacetylate HIF-1α, resulting in the production of VEGF-A and VEGF-C, which are linked to lymphangiogenesis [51] and metastasis [17, 50].

Ohshiro et al. examined if MTA1 controls the expression and activities of estrogen receptor β (ERβ)—a potent tumor suppressor. In the HSG and HSY salivary duct carcinoma cell lines, they discovered that endogenous MTA1 depletion increases ERβ expression. Additionally, MTA1 knockdown prevented HSG and HSY cells from proliferating and invading. Since a proteasome inhibitor might stop it, the observed ERβ downregulation by MTA1 overexpression involves the proteasomal degradation process. Furthermore, in both cell lines, ERK1/2 signaling was reduced, and cell migration was slowed by both MTA1 knockdown and ERβ overexpression. According to these findings, MTA1 dysregulation in a subset of salivary gland cancer may compromise ERβ's tumor suppressor function, leading to aggressive phenotypes. As a result, the MTA1-ERβ axis may be a novel therapeutic target for salivary gland cancer [52].

On the other hand, Andisheh-Tadbir et al. [17] found no statistically significant association between the expression of MTA1 protein and the clinical stage, the status of lymph nodes, or the metastasis of salivary mucoepidermoid carcinoma. This discovery could be attributed to insufficient cases or the association between tumor growth and positive lymph node metastasis and small sample size [17]. However, MTA1 immunoreactivity was found to be significantly higher in malignant tumors compared to benign tumors, as well as higher in benign tumors compared to normal salivary gland tissues (nSGTs). This finding was made by Andisheh-Tadbir et al. [17]. This finding implies that MTA1 is involved in the development of salivary gland tumors and reflects the aggressiveness of these malignant tumors in comparison to pleomorphic adenoma [17]. The elevated MTA1 levels seen in ovarian cancer in comparison to normal ovarian epithelium are in line with these findings [20].

Li et al. showed that MTA1 overexpression is linked to lymph-node metastases and TNM staging in non-small-cell lung carcinoma rather than the clinical and histological subtypes of the tumor [53]. Although there was no significant correlation found between the histology and clinical status, the authors of another study showed that MTA1 overexpression in esophageal cancer was linked to T-status [54].

In the present study, a strong positive correlation between PROX1 and MTA1 expressions was found that may be supported by the evidence that prox1 and MTA1 trigger and control the EMT which is considered the most crucial phenomenon in cancer progression and metastasis [27, 30].

In the current study, the assessment of the prognostic significance of PROX1 and MTA1 revealed that, in the univariate models, high MTA1 expression and positive PROX1 expression were predictive variables for poor DFS and OS in this sample of patients. Furthermore, cases with large-sized tumors (T3 + T4), positive nodal involvement, positive distant metastases, advanced TNM clinical stage (III + IV), high-grade carcinomas, presence of LV invasion, and old age (≥ 65 years) had significantly lower DFS and OS (*P* values < 0.05).

To the best of our knowledge, this is the first study to assess the predictive significance of MTA1 and PROX1, as well as their relationship to OS and DFS in malignant salivary gland tumors. Moreover, an additional study assessed the OS of patients with squamous cell carcinoma (SCC) and found that patients with elevated MTA1 levels had a worse prognosis [41]. MTA1 was found to be overexpressed in highly metastatic cells across a range of malignancies in other investigations [55, 56]. Therefore, there seems to be a correlation between high MTA1 levels and poor survival results.

Only PROX1 expression was shown to be a negative predictive factor for DFS in the multivariate analysis [32]. In a study by Rodrigues et al., the predictive value of PROX1 in oral SCC was found to be contrary. More precisely, compared to OSCC, PROX1 expression levels were much greater in non-neoplastic margins, and there was no significant association between PROX1 expression and any clinicopathological factors [35].

When comparing normal gastric mucosa and non-metastatic LN tissues to gastric cancer samples and metastatic LNs, Park et al. discovered elevated expression of PROX1. Individuals who had PROX1-positive tumors had a higher probability of dying and a noticeably lower OS [11]. The study by Ueta et al. also reported a link between PROX1 overexpression and a worse prognosis in gastric cancer cases. Compared to their low-PROX1-expressing cases, patients with high PROX1 expression had a significantly worse 5-year OS and a shorter recurrence-free survival rate [57].

Consequently, PROX1 and MTA1 are regarded as useful biomarkers for the diagnosis, prognosis, and treatment of malignancies in humans; nevertheless, additional research is required to thoroughly evaluate their function in cancer therapy and their potential clinical application.

## Conclusion

PROX1 and MTA1 have the potential to be utilized as predictors of progression and recurrence in SGCs because they are good indicators of LVI, recurrence, distant metastasis, and tumor stage. In SGCs, PROX1 and MTA1 represent viable molecular therapy targets as well as potentially useful prognostic and predictive biomarkers.

### Limitations of the study

Although immunohistochemistry provided valuable findings in evaluating PROX1 and MTA1 expression at different histologic grades of SGCs, utilizing other techniques such as western blotting, in situ hybridization, or PCR could enrich the study. Furthermore, even though the worked sample is thought to be somewhat small, it is still legitimate and can offer insightful information. We, therefore, advise using a bigger sample size in future research to generalize the findings.

## Data Availability

No datasets were generated or analysed during the current study.

## Abbreviations

SGCs: Salivary gland carcinomas
PROX1: Prospero homeobox 1
MTA1: Metastasis tumor antigen 1
ADCC: Adenoid cystic carcinoma
CXPA: Carcinoma ex pleomorphic adenoma
MEC: Mucoepidermoid carcinoma
ACC: Acinic cell carcinoma
LVI: Lymph-vascular invasion
DM: Distant metastasis
MSG: Major salivary gland
IHC: Immunohistochemistry
