# Correction: Prognostic relevance of prospero homeobox 1 and metastasis tumor antigen 1 in patients with malignant salivary gland tumors: a clinicopathological study

**DOI:** 10.1186/s40001-025-03682-1

**Published:** 2026-01-27

**Authors:** Samar Soliman, Doaa Abdallah Farag, Heba Ahmed Elhendawy

**Affiliations:** Oral Pathology, Faculty of Dentistry, Mansoura University, Mansoura, Egypt

**Correction: European Journal of Medical Research (2025) 30:622** 10.1186/s40001-025-02863-2

In this article, the p-value notation '0.000' should have read 'p < 0.001'.

